# Process-Driven Modelling of Media Forensic Investigations-Considerations on the Example of DeepFake Detection

**DOI:** 10.3390/s22093137

**Published:** 2022-04-20

**Authors:** Christian Kraetzer, Dennis Siegel, Stefan Seidlitz, Jana Dittmann

**Affiliations:** Department of Computer Science, Otto-von-Guericke University, 39106 Magdeburg, Germany; dennis.siegel@ovgu.de (D.S.); stefan.seidlitz@ovgu.de (S.S.); jana.dittmann@iti.cs.uni-magdeburg.de (J.D.)

**Keywords:** media forensics, forensic process model, certifiable investigation methods, DeepFake detection

## Abstract

Academic research in media forensics mainly focuses on methods for the detection of the traces or artefacts left by media manipulations in media objects. While the resulting detectors often achieve quite impressive detection performances, when tested under lab conditions, hardly any of those have yet come close to the ultimate benchmark for any forensic method, which would be courtroom readiness. This paper tries first to facilitate the different stakeholder perspectives in this field and then to partly address the apparent gap between the academic research community and the requirements imposed onto forensic practitioners. The intention is to facilitate the mutual understanding of these two classes of stakeholders and assist with first steps intended at closing this gap. To do so, first a concept for modelling media forensic investigation pipelines is derived from established guidelines. Then, the applicability of such modelling is illustrated on the example of a fusion-based media forensic investigation pipeline aimed at the detection of DeepFake videos using five exemplary detectors (hand-crafted, in one case neural network supported) and testing two different fusion operators. At the end of the paper, the benefits of such a planned realisation of AI-based investigation methods are discussed and generalising effects are mapped out.

## 1. Introduction

Modern day media forensics is a strongly pattern recognition, respectively, artificial intelligence (AI) driven domain. In a recent white paper titled “Secure, robust and traceable use of AI-problems, procedures and actions required” [1] (translated from the German title “*Sicherer, robuster und nachvollziehbarer Einsatz von KI-Probleme, Maßnahmen und Handlungsbedarfe*”), the German Federal Office for Information Security (BSI, the German national cybersecurity authority; Since forensics, as part of legal proceedings, is regulated on basis of national legislation, research in forensics also has to acknowledge national legal and statutory requirements – in the case of this paper, therefore, besides internationally accepted best practices, like the Daubert standard (see Section 2), the German national situation is reflected, due to the fact that all authors are working at a German research institution and the work is funded in part by the German Federal Ministry of Education and Research (BMBF)) summarises the current situation with regards to trustworthy and reliable AI applications as follows: There is currently an urgent need for further research into the security of AI systems, in order to be able to make reliable statements about the security and confidence of such systems. According to the BSI, there are three specific aspects on which research needs to focus:

1. Development of standards, technical guidelines, test criteria and test methods: Currently, there exist no such standards that are sufficiently suitable for assessing the security and reliability of AI systems for critical contexts (such as health care, finance health care, finance, etc.). There is also a lack of security benchmarks for less critical applications (with a few exceptions).
2. Research effective countermeasures against AI-specific attacks: The existing measures for such attacks are often insufficient. In order to ensure a secure and robust operation of AI systems, further countermeasures must be researched.
3. Research into methods of transparency and explainability: The often inadequate explainability of AI systems has a significant influence on their Information Technology (IT) security and causes a lack of acceptance of the systems.

What holds true for every form of AI usage is even more important if it comes down to AI-driven processes that are (by regulation) restricted to decision support systems, e.g., in the case of forensics, where it internationally accepted standard that investigation results have to be interpreted in expert testimony. Here, the corresponding expert has to be able to explain the investigation method as well as all aspects influencing an investigation outcome in front of a trier of fact (in most cases a single judge, a group of judges or a jury). Besides other reasons, this human presentation and interpretation is considered necessary because the expert can also interpret contextual information to reason about the intention of an action (e.g., why a DeepFake video has been created—see Section 2.2 for a list of white hat application scenarios for this dual-goods media manipulation method), which is a challenge where the AI alone will still fail.

As **contributions of this paper**, the following items are addressed:

- The need for modelling forensic processes is reasoned upon.
- A concept for modelling media forensic investigation pipelines is derived from established guidelines.
- The applicability of such modelling is illustrated on the example of a media forensic investigation pipeline focusing on the detection of DeepFake videos. It is important to already mention at this point, that the DeepFake detectors, test criteria and test methods used in this paper are used for illustrative purposes on the processes and are **not** claiming to represent the state-of-the-art in detector research.
- The benefits of such a planned realisation of AI-based investigation methods are discussed.

Regarding the first of these items (the reasoning on the need for modelling forensic processes) it is shown that forensic process models are an important cornerstone in the science and more importantly the practice of forensics. They guide investigations and make them comparable, reproducible as well as certifiable. Usually, the adherence to strict guidelines (i.e., process models) are regulated within any legal system (e.g., in the US by the fourth of the Daubert criteria (“*the existence and maintenance of standards and controls*” [2])). For mature forensic sciences, like for example fingerprint analysis, internationally accepted standards (like the Analysis, Comparison, Evaluation and Verification methodology (ACE-V) process model for dactyloscopy) have been established over the last decades. Due to the fact that IT forensics is a rather young discipline in this field (with media forensics being an even younger sub-discipline), it is hardly astonishing that here the forensic process models have not yet achieved the same degree of maturity as in other fields. For this reason, an effort is made here to move this field forward by presenting a concept for modelling media forensic investigation pipelines, which is derived from well-established guidelines. Since all the authors are working at a German research institution, here an extension of the guidelines on IT forensics [3] by the German Federal Office for Information Security (BSI) is used as the basis for this work.

Regarding the third item from the list of contributions identified above, the applicability of the proposed modelling work is illustrated on the example of a media forensic investigation pipeline focusing on the detection of DeepFake videos. This application scenario it chosen because it is a recent threat scenario that currently achieves a lot of research attention due to the potential implications it has for the trust assumptions in video material used (amongst other scenarios) in political debates. Here, an already complex investigation pipeline taken from previous work [4] consisting of three detectors plus a fusion operator (with two alternative fusion methods tested) is extended by adding two additional detectors. Despite the fact that both new detectors are performing in benchmarking significantly better than guessing (with a Kappa value of κ∼0.4), the following empirical evaluations show a slight drop in the average detection performance (at least by κ=0.025). This drop is neither the expected nor an intuitive outcome, but illustrates how important an extensive benchmarking of methods prior to field application (also in a fusion setup) is. While the detection methods used here are admittedly not amongst the most sophisticated detectors currently available, their general performance, especially the mentioned problems with the generalisation power, are representative for the current situation in this field of applied pattern recognition.

Following the discussions on this empirical work, the benefits of such a planned realisation of AI-based investigation methods are discussed in the contexts of development of standards, technical guidelines, test criteria and test methods on one hand and research into methods of transparency and explainability of AI methods on the other hand.

The rest of the paper is structured as follows: In Section 2, a brief overview on the state of the art on forensic process modelling for media forensics and DeepFake detection is presented. This is followed in Section 3 by a summary of related work aiming at advancing the basic forensics guidelines used in this paper (here, the German BSI guidelines on IT forensics). Based upon these foundations, Section 4 introduces the modelling work in this paper (based on previous work in Siegel et al. [5]). This chapter also summarises known evaluation best practices, metrics as well as DeepFake data sets. In Section 5, an application example using components from the introduced process modelling is given for the description of a fusion-based DeepFake detector pipeline. The descriptions are divided into a planning/templating phase and the instantiation of the pipeline for all evaluations in this paper. Section 6 provides a brief summary of the results, before the following Section 7 projects the conclusions onto the contributions identified in Section 1. The paper is concluded by a short view into potential future work in Section 8.

## 2. State of the Art on Forensic Process Modelling for Media Forensics and DeepFake Detection

In a very recent textbook on media forensics targeting digital face manipulations [6], the authors reflect the current academic perspective on media forensics as: “*In case manipulation detection methods are used by public authorities competent for preventing, investigating, detecting, or prosecuting criminal offences this shall be done in a lawful and fair manner. While these are broad concepts, case law further explains how to apply these concepts*.” Those mentioned characteristics are further specified in [6] as:

- Lawfulness: “*refers to the need* […] *to adopt adequate, accessible, and foreseeable laws with sufficient precision and sufficient safeguards whenever the use of the detection technology*, […], *could interfere with fundamental rights and freedoms*”.
- Fairness: “*points to the need for being transparent about the use of the technology. Furthermore, it is obvious that the use of the detection methods should be restricted to well-defined legitimate purposes*, […]”.

Regarding the fairness, the authors in [6] point out that when intended for court usage, explainability of the forensic algorithms used is a strong requirement. In addition, they state that: “*From an organizational point, one should also know that decisions purely and solely based on automated processing, producing adverse legal effects or significantly effecting subjects, are prohibited, unless authorized by law, and subject to appropriate safeguards, including at least human oversight and intervention*.”

In accordance with other well established works originating in the academic parts of media forensics research (like [7]), the synopsis presented in [6] is that “[*t*]*he absence of a unified approach, common regulatory framework, and commonly accepted practices has resulted in a situation where different initiatives emerge across countries which share some common elements but also numerous differences that can lead to challenges related to interoperability*”.

An important step towards more mature forensics are forensic process models. They guide investigations and are supposed to make them comparable, reproducible, as well as certifiable. Usually, the adherence to strict guidelines (i.e., process models) are regulated within any legal system (e.g., in the US by the fourth of the Daubert criteria (“*the existence and maintenance of standards and controls*” [2])).

Due to the fact that IT forensics is a rather young discipline in this field (with media forensics being an even younger sub-discipline) it is hardly astonishing that here the forensic process models (if they exist at all) have not yet achieved the same degree of maturity as in other fields. Nevertheless, they would still be important to achieve universal court acceptability of methods.

To pay respect to the difficulties in this domain, the following two subsections provide the following: A brief overview over forensic process modelling requirements and best practices for media forensics are presented in Section 2.1, starting with an international perspective and then narrowing down for the German perspective relevant for the authors of this paper. These discussions are then followed in Section 2.2 by a brief summary on the current state of the art in the application domain of DeepFake detection, which is the chosen application scenario within this paper.

### 2.1. Forensic Process Modelling for Media Forensics

In contrast to the international perspective of academic research on media forensics, its field application is governed by national legislation. Undeniably the most active judicial system worldwide, with a high demand for forensic and media forensic investigations, is found in the USA. Naturally, a well-established set of best practices is the result. In Section 2.1.1, a very brief overview on these best practices is presented. In the following Section 2.1.2, the German situation, relevant to the authors, is reflected.

As a preamble to this section, it has to be highlighted that all authors are computer scientists and possess absolutely no legal training. All statements and interpretations presented below on legal considerations are therefore layman’s interpretation of freely available material, which are made to the best of the authors’ knowledge.

#### 2.1.1. Forensic Process Modelling Requirements and Best Practices (US Perspective)

On the U.S. federal level, strict rules for the integration of the results of forensic investigations were established in 1975. These rules, the Federal Rules of Evidence (FRE [8]), define the framework within which evidence can be admitted into court. Even if these rules are in their original form only applicable on U.S. federal level, their concepts for handling forensic data have influenced many other judicial systems worldwide and are also considered with interest in many European legal systems (see [2]).

In general, under the FRE, forensic results have to be interpreted by experts to the court. The reason for this lies in the assumption that any judge (or jury) will lack the expert knowledge to completely interpret the findings of a forensic investigation on his/her own and that therefore expert testimony is strictly required in court proceedings. If the expert’s opinion helps the fact finder in understanding the significance of factual data, then the expert witness is essential for the case and its opinion evidence is admissible.

Using the terminology of U.S. jurisdiction, the trial judge acts as a form of ‘gatekeeper’, assuring that scientific expert testimony truly proceeds from reliable (or scientific) knowledge. Considerations on relevance and reliability require the trial judge to ensure that the expert’s testimony is ‘relevant to the task at hand’ and that it rests ‘on a reliable foundation’. According to [9], the primary rules that are relevant for the presentation of forensic evidence in court (i.e., that apply to expert witnesses) in the FRE are FRE rule 702 (“*Testimony by Experts*”) and FRE rule 703 (“*Bases of Opinion Testimony by Experts*”).

In the year 2011, FRE rule 702 (“*Testimony by Experts*”) was amended to: “*A witness who is qualified as an expert by knowledge, skill, experience, training, or education may testify in the form of an opinion or otherwise if: (a) the expert’s scientific, technical, or other specialized knowledge will help the trier of fact to understand the evidence or to determine a fact in issue; (b) the testimony is based on sufficient facts or data; (c) the testimony is the product of reliable principles and methods; and (d) the expert has reliably applied the principles and methods to the facts of the case*”.

When analysing this rule, it can be seen that, in regarding the admissibility of an expert, the judge has to establish whether the following four points are met:

- **Qualification of a witness as expert:** First, a witness has to qualify as an expert. The conclusion of this process is that the presiding judge decides whether the witness may offer opinion testimony as an expert.
- **Type of knowledge considered:** The first seven words of FRE rule 702 specify different types of knowledge (e.g., scientific, technical or other specialised knowledge) that an expert can offer.
- **Who is addressed by the expert:** Basically, there are two entities the expert has to convince. First, the judge, to get admitted in pre-trial hearings, and second the ‘fact finder’ (the “*trier of fact*” in FRE rule 702 [10], either a jury in normal cases or a judge in non-jury trials) at the trial itself.
- **Qualification:** Any expert has to testify upon the five criteria listed in FRE rule 702 “*knowledge, skill, experience, training, or education*” [10]. This information helps the judge to decide whether an expert can be admitted to trial in a specific case and helps the ‘fact finder’ (i.e., usually the jury) to assign corresponding weights to each expert’s testimony in the decision process.

If these four points are established, the judge determines for the case whether an expert is qualified to testify under FRE rule 702. The April 2000 (effective December 2000) amendment of FRE rule 702 includes three further requirements, which must also be met. The goal of these additional requirements is to make it easier to present effective scientific and technical expert testimony whenever such evidence is warranted and provide a basis for the exclusion of opinion testimony that is not based on reliable or mature methodology. These additional requirements are [10]: “[…] *if (1) the testimony is based upon sufficient facts or data, (2) the testimony is the product of reliable principles and methods, and (3) the witness has applied the principles and methods reliably to the facts of the case*”. In April 2011, another requirement was added to this list [8] “[…] *the expert’s scientific, technical, or other specialized knowledge will help the trier of fact to understand the evidence or to determine a fact in issue* […]”.

In the notes on FRE rule 702 published by the Legal Information Institute at Cornell Law School in December 2010 [11], the current regulations regarding the interpretation of this rule for U.S. federal courts are summarised as follows: “*Rule 702 has been amended in response to* Daubert v. Merrell Dow Pharmaceuticals, Inc., *509 U.S. 579 (1993), and to the many cases applying Daubert, including* Kumho Tire Co. v. Carmichael, *119 S.Ct. 1167 (1999). In Daubert the Court charged trial judges with the responsibility of acting as gatekeepers to exclude unreliable expert testimony,* […]”. The main result of this amendment are the so called Daubert hearings where the judge(s) are supposed to use the so called Daubert criteria (see below) to assess the admissibility of methods and investigation results to legal proceedings.

The other FRE regarding opinions and expert testimony (rule 701 “*Opinion Testimony by Lay Witnesses*”, rule 703 “*Bases of an Expert’s Opinion Testimonies*”, rule 704 “*Opinion on an Ultimate Issue*”, rule 705 “*Disclosing the Facts or Data Underlying an Expert’s Opinion*” and rule 706 “*Court-Appointed Expert Witnesses*”; see [8]) are further regulating the usage of forensic investigation results in court, but are of little relevance to this paper. For a more detailed analysis, see [12].

Regarding the second and third point of the list given above in the analysis of FRE rule 702 (‘Type of knowledge considered’ and ‘Who is addressed by the expert’), it has to be summarised that if something is declared to be ‘science’ in regard to FRE rule 702, then the criteria for the evaluation of scientific methods introduced in *Daubert v. Merrell Dow Pharmaceuticals, Inc.*, 509 U.S. 579 (1993) [13], ref. [14] have to be applied by the judge to make the expert prove this declaration.

In 1923, the court in *Frye v. United States*, 293 F. 1013 (D.C. Cir. 1923) made a first suggestion how to proceed with the admission of expert testimony based on novel forensic techniques. The court in Frye suggested [15]: “*Just when a scientific principle or discovery crosses the line between the experimental and demonstrable stages is difficult to define.* […], *the thing from which the deduction is made must be sufficiently established to have gained general acceptance in the particular field in which it belongs*”. In Frye (or the Frye standard as it is also referred to) the court concluded that the polygraph test that was intended to be used in this case could not be admitted because it lacked the required general acceptance in the corresponding research fields. Prior to this seminal ruling in Frye, according to [9], the competence of an expert was equivalent to his success in real life. In [9] it is summarised as: “*If a person earned a living selling his or her knowledge in the marketplace, then that person would be considered an expert who could testify at trial.*”

The Frye standard was in 1975 partially replaced by the FRE. Initially, they contained no special rule that, when dealing with ‘scientific’ evidence, novel or otherwise, ensured that science-based testimony is reliable and, therefore, admissible. Therefore, all evidence was considered admissible if relevant, provided its use in court was not outweighed by “*unfair prejudice, confusing the issues, misleading the jury, undue delay, wasting time or needlessly presenting cumulative evidence*”, as stated in FRE rule 402 [8].

The next relevant step in legal developments on expert testimony (and therefore the means of introducing forensic sciences into court) occurred in 1993, when the U.S. Supreme Court made another ground-breaking decision on expert testimony in *Daubert v. Merrell Dow Pharmaceuticals, Inc.*, 509 U.S. 579 (1993) [13]. Daubert was in 1999 followed by another important court case, *Kumho Tire Co. v. Carmichael*, 119 S.Ct. 1167 (1999). Both Daubert and Kumho Tire arose out of civil lawsuits. An extensive and intelligible summary of the proceedings in the Daubert cases (original and the affirmation in the U.S. Court of Appeals) is presented in [9]. The main point of interest for this paper is that the court unanimously held that Frye did not survive the enactment of the FRE. In interpreting FRE rule 702, the court in Daubert stated that if the admissibility of scientific evidence is challenged, it is the function of the trial court to act as ‘gatekeeper’ to determine whether proffered opinion evidence is relevant and reliable. The U.S. Supreme Court specified several flexible and non-exclusive criteria (the so-called Daubert criteria or Daubert standard) to guide other courts when they have to consider in deciding whether a scientific field is sufficiently reliable to warrant admission of opinion evidence. As a further important milestone, in 1999 in *Kumho Tire Co. v. Carmichael*, 119 S.Ct. 1167 (1999), the U.S. Supreme Court applied the Daubert criteria of proof of reliability to all forms of expert opinion testimony (i.e., scientific, applied science, technological, skill and experience). Additionally, the court in Kumho Tire made it clear that the list of Daubert criteria was meant to be helpful and is not a definitive checklist, but rather a flexible, non-exclusive recommendation. As a result, no attempt has been made in US law to ‘codify’ these specific criteria. Other U.S. law cases have established that not all of the specific Daubert criteria can apply to every type of expert testimony. The specific criteria, explicated by the Daubert court, are [11]:

“*whether the expert’s technique or theory can be or has been tested – that is, whether the expert’s theory can be challenged in some objective sense, or whether it is instead simply a subjective, conclusory approach that cannot reasonably be assessed for reliability*”;

“*whether the technique or theory has been subject to peer review and publication*”;

“*the known or potential rate of error of the technique or theory when applied*”;

“*the existence and maintenance of standards and controls*”;

“*whether the technique or theory has been generally accepted in the scientific community*”.

While the criteria DC2 to DC5 are self-explanatory (including the fact that publication in DC2 means ‘open publication’), DC1 is summarised more precisely in [13] as “*the theory or technique (method) must be empirically testable, falsifiable and refutable*”.

The Daubert criteria are widely accepted in the classical fields, like medical forensics. It can also be, and is, applied in the much younger field of IT-forensics (see e.g., [16,17]). It has to be admitted that the field of media forensics, which is the focus of this thesis, is still lacking maturity in this regard. Here, only very specific methods applied in this field already fulfil the Daubert criteria sufficiently. Overviews over the more mature techniques in this field are given in [18,19].

A well-established reference in this field is the document Forensic Examination of Digital Evidence: A Guide for Law Enforcement [20] of the U.S. Department of Justice-National Institute of Justice. Unfortunately, this document has not received any update since 2004. Its place has been taken over in past years by publications of well-established (and court-trained) forensic experts, such as [21,22] or [23]. Homogenising the different individual views, expert bodies, like the Organization of Scientific Area Committees (OSAC) Task Group (TG) on Digital and Multimedia Evidence have become normative institutions arguing for harmonisation of procedures: “[…]*digital/multimedia evidence, and other forensic disciplines, would be in a much stronger position to demonstrate their scientific basis if they were considered as belonging to a harmonized forensic science rather than as mere disciplines at the intersection of forensic specialties and other sciences*”. [24]. As a reason, the following is given: “*Like many other specializations within forensic science, the digital/multimedia discipline has been challenged with respect to demonstrating that the processes, activities, and techniques used are sufficiently scientific*”. This OSAC TG aims at advancing digital/multimedia evidence, and forensic science as a whole by (amongst other aspects):

“*Strengthen scientific foundations of digital/multimedia evidence by developing systematic and coherent methods for studying the principles of digital/multimedia evidence to assess the causes and meaning of traces in the context of forensic questions, as well as any associated probabilities*.”

“*Assess ways to mitigate cognitive bias in cases that require an understanding of the context of traces in order to analyze digital/multimedia evidence,* […]”

“*Establish effective ways to evaluate and express probative value of digital/multimedia traces for source level and activity level conclusions. This includes studying how quantitative evaluation of digital/multimedia evidence can be constructed for different forensic questions,* […] *as well as studying how such evaluative results can be communicated to decision-makers.*”

As a consequence, generalisable and standardised forensic process models are currently sought for to bridge the gap between the strict legal requirements (see the FRE 702 and Daubert requirements discussed above) and the current degree of (or rather lack of) maturity of many media forensic approaches originating form academic research.

#### 2.1.2. The German Perspective

As discussed in detail in [2], the situation in the U.S. can not be directly projected onto the European situation. One of the main reason is that forensics are still entirely governed by national legislation.

For the authors the German situation is relevant. Here, the currently most relevant official guideline is the BSI code of practice for IT forensics (“Leitfaden IT-Forensik” [3]) of the German Federal Office for Information Security (BSI). One of the intentions of this document was to try to homogenise forensic proceedings in the highly fragmented system with 35 different police agencies independent from each other on federal- and state level. In this regard, it is very similar in its intention to the document Forensic Examination of Digital Evidence: A Guide for Law Enforcement [20] (2004) of the U.S. Department of Justice-National Institute of Justice and similar to its U.S. pendant, it is outdated with the last updated version of the “Leitfaden” (German for guidelines) having been published in 2011. Nevertheless, it is still a valuable starting point and has been used as such for more recent work on forensic process modelling, see Section 3 below.

**Figure 1:**
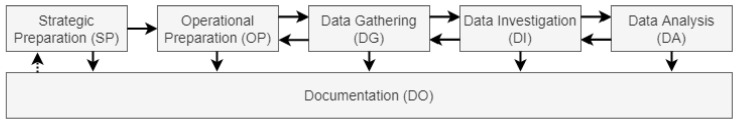
Phase model (based on [3]), extended to include an optional feedback loop from the Documentation (DO) into the strategic preparation (SP).

In its core, the BSI guidelines for IT forensics define a phases driven process model model, tool categories and a forensic data model. In the phase driven process model, which is for this paper the most relevant component of these guidelines, six different phases are described: Strategic preparation (SP), Operational preparation (OP), Data gathering (DG), Data investigation (DI), Data analysis (DA) and Documentation (DO). These phases, which are outlining the process itself, are briefly summarised in Table 1 the interaction pattern of these phases is shown in Figure 1. The actual passing of data and results between the phases is taking place in the horizontal transitions, shown as horizontal arrows in the figure. It has to be admitted here, that this paper somewhat diminishes the role the Documentation receives in [3]. Originally, the DO is considered to have two distinguishable aspects: the accompanying documentation of the process (which can be seen as a combination of complete logs as well as a tamper-proof (hence the uni-directional, solid-lined vertical arrows in the figure), digital chain-of-custody) and the final documentation (e.g., as the written expert report intended to be used in court as basis for an expert testimony). In the present context, it is important to point out that the latter (i.e., the drafting of the final documentation for a case) should be used to reflect upon potential improvements of the processes and their implementation, acting as a feedback loop into SP. This is shown in Figure 1 by adding the dashed arrow from DO into SP.

One important aspect here is the separation of preparation steps in an investigation into two distinct phases (the strategic preparation (SP) on one hand, and the operational preparation (OP) on the other). In recent work on this model (e.g., [25], which is available in English), the SP is generally defined as: “*The strategic preparation* […] *includes all preparation procedures taken ahead of the actual occurrence of a specific incident*”. Exemplary measures for SP in the context of digital forensics are given by [25] as: “*Documentation and extension of knowledge of IT systems specifics, tool testing for forensic data types and sets of methods determination for error loss and uncertainty estimation, setup of logging capabilities, performance of system landscape analysis, data protection considerations,* […].” In contrast, the OP is specified to “[…] *include all preparation procedures taken after of the actual occurrence of a specific incident. Those procedures by definition do not alter any data on the targeted system*”. These preparation phases are then followed by the actual application of forensic procedures, which can be separated into the triplet of data gathering (DG), data investigation (DI) and data analysis (DA). The whole process is in every phase (including SP and OP) supported by accompanying documentation, which is in the last phase (documentation (DO)) used as the basis for the generation of the official documents regarding the investigation (e.g., the evidence to be interpreted in expert testimony in a court case). It has to be acknowledged here that these BSI guidelines on outlining a forensic process, while acknowledging established best practices in this field, significantly differ from other national guidelines, even in other EU states. This can be illustrated by comparing it, for example, with the model described in [27], which very well reflects the Norwegian approach. It also builds upon a phase-driven model, but with a different established phases layout: (1) Identification Phase, (2) Collection Phase, (3) Examination Phase, (4) Analysis Phase and (5) Presentation Phase. This is much closer to long-time established best practices in traditional (analogue world) forensic sciences and requires then explicit activities to achieve and maintain “Digital Forensic Readiness” [27] (an equivalent to the Strategic Preparation phase in the BSI guidelines) to successfully cope with modern day digital and digitised forensics tasks.

The second core aspect of the BSI guidelines is the classification scheme for forensically relevant data types. More recent publications (see Section 3 below) have shown that the original scheme as proposed by the BSI in 2011 needs to be extended accordingly if investigation domains other than hard-disk, RAM or network forensics are considered.

The third core aspect of the BSI guidelines is the definition of forensic method classes. For a detailed discussion on these method classes, including considerations on the availability in certain investigation contexts, practicalities of their application in a forensic process, etc., we refer to [25].

### 2.2. (Brief) Summary on the Domains of DeepFake Generation and Detection

The methodologies and solution concepts for the generation of DeepFake material are manifold. Due to this reason, these generative processes (which are mostly outside the scope of this paper) are covered extensively in survey publications, like [28] or the corresponding chapters in [6]. Generally, they are divided into the classes of *facial re-enactment*, *facial replacement* (or face swapping), *face editing* and *face synthesis*.

If a target persons facial expression corresponds to the expression of another person, presented as controlling input, then the generation process is called as facial re-enactment. For the case of face replacement, a source face is transferred to a face in a target media object where the facial expression of the target person has not changed. Face editing addresses the same face in an image or video. Only the facial expressions or some face parts are modified. In contrast to the methodologies described above, face synthesis refers to newly created faces that are not linked to real persons [28].

The usage of DeepFakes does not automatically imply black hat (i.e., malicious) applications, but also a large number of white hat (i.e., benign or non-malicious) application scenarios exist. The following subSection 2.2.1 briefly summarises examples for both types of application scenarios, using the four different classes of generative processes mentioned above.

The different generation strategies also create class-related artefacts in the output media objects. In Section 2.2.2, these are very briefly summarised.

#### 2.2.1. DeepFake Use Cases

The use of DeepFakes has a wide range of possible application scenarios, where their impact can be on an individual or societal level. While mainly the negative aspects are highlighted in existing literature, there are also positive examples of the use of DeepFakes. As stated in [6]: “[…] *it is important to note that face manipulation techniques are also expected to have positive impact on society and economy*. […] *can help to address privacy issues through privacy-enhancing techniques, they facilitate the training of machine learning models with synthetic data* […], *they can help with sustainability by facilitating virtual fitting rooms for the beauty and fashion industries and drive economic development with (high added value) mobile e-commerce, entertainment, and social media applications*.”

This non-exhaustive list can easily be extended, with most use cases having both positive and negative aspects to be considered. DeepFakes have received first news coverage due to usage in pornographic contexts using face-swaps, where primarily women became victims of targeted defamation. Face-swaps are also used for white hat applications, e.g., showing the user wearing certain clothes (‘magic mirror’ scenarios for online shopping). In the context of lip synchronisation techniques used in DeepFakes, the most prevalent examples show the manipulation of video footage and the spoken word of well known politicians (e.g., former US president Barack H. Obama or Nancy Pelosi in her time as Speaker of the United States House of Representatives), to spread misinformation. On the other hand, the same technique can be used to break language barriers, in the example of the “Malaria Must Die” campaign, where the famous football star David Beckham addresses the audience in this health campaign in nine languages, due to the help of DeepFake technology. In addition to the use of real voices, the use of synthetic voices is also an application scenario to be discussed here. In white hat applications, this can increase the accessibility of content (e.g., in text-to-speech systems). A possible threat of this synthetic voice (or rather imitation of an existing voice) are the so-called Vishing attacks [29]. In terms of face editing, the main purpose is fun applications, e.g., to simulate ageing, different hair styles and makeup. Although the authors are not aware of any attack scenario based on face editing, its use for rejuvenation and artificial ageing could pose a challenge for youth protection. Another well-used application is the fictitious resurrection of deceased people, which is often used to retain established actors for cinematic productions (e.g., Peter Wilton Cushing in the film “Rogue One”). It can also be used for a more immersive experience in education or to provide a more immersive experience in education (e.g., historical facts presented by a contemporary witness). While the intents are positive, the use results both in ethical and legal questions.

Finally, it is important to note that AI cannot decide whether a DeepFake is used positively or negatively. A human observer/expert is always needed here to decide between black hat and white hat application, based on the context of the usage of DeepFake technologies as summarised above.

#### 2.2.2. DeepFake Detection

Because of their creation process, most DeepFakes are inherently compromised with artefacts or traces which might unmask fake media. The amount and type of those artefacts are versatile and depend on the used creation method. Artefacts are divided primary into visual artefacts within single video frames (intra-frame) and temporal artefacts across several video frames (inter-frame) [30]. Furthermore, Mirski et al. [28] subdivide both those categories in smaller artefact categories. In case of visual artefacts, they differ between blending, environment and forensics. Blending refers to “*generated content* [which] *is blended back into the frame*” [28]. Blending artefacts are marked by edges. Environment artefacts are specified by the content which differs from the rest of the frame (e.g., different lightning conditions). Forensic artefacts are special fingerprints which are created by DeepFake generation models (e.g., Convolutional Neural Networks (CNN)). Additionally, imperfections like unnatural head poses are mentioned in this context. Temporal artefacts are distinguishing between behaviour, physiology, synchronisation and coherence. Regarding behaviour artefacts, it is easier to replace one face by another than to copy the (gestical) behaviour of the person. The investigations of similar but also different behaviours could be a hint to those DeepFake artefacts. With a specific video camera setting, it is possible to detect physiological signals like the heart rate of a person. Currently, DeepFake videos are not able to reproduce these physiological signals before those physiological artefacts are indications for interferences in DeepFake videos. Synchronisation artefacts address inconsistencies between lip movements and the corresponding voice. Coherence artefacts describe, e.g., flickers and jitters which may be present in DeepFakes [28].

Many different approaches detect those different artefacts with varying detection methods, which can be ordered into the following two main groups: Hand-crafted and learned feature methods. Most approaches detect DeepFake artefacts with neural networks and a huge amount of example data. After many training iterations, they analyse the example data and produce learned features which are needed for further classification steps. Convolutional Neural Networks (CNNs) are able to detect spatial features whereas Recurrent Neural Networks (RNNs) are preferable for the detection of temporal features. Li et al. [31] detect eye blinking with a Long-term Recurrent Convolutional Network (LRCN) model, which consists mainly of three parts: feature extracting, sequence learning and state prediction. They also suggest this approach for a DeepFake detection. However, the paper of Li et al. [31] does not goes into detail in case of the evaluation of DeepFake detection.

In contrast to the neural networks-based methods, the alternative approach is learning to identify DeepFake material with pre-defined, hand-crafted features defined by domain experts. Hand-crafted feature methods are in DeepFake detection less common and of these few existing papers using hand-crafted methods, like [32,33,34], most detect DeepFake videos using Support Vector Machines (SVMs), typical 2-class classifiers. Other hand-crafted feature methods (e.g., [4]) are implemented by decision trees. Jung et al. [35] created a detector called DeepVision based on the Eye-Aspect-Ratio (EAR) of Soukupov et al. [36], which combines Machine Learning techniques with heuristic methods based on results of medical-, biology- and brain engineering research. They used the knowledge of the behaviour of human eye blinking for the detection approach of their DeepFake detector. Nevertheless, they tested their approach in [36] only on a statistically insignificant number of different DeepFake videos (without any attempt to also determine the amount of false positive errors on benign material).

Three of the five detectors used for empirical experiments in this paper are re-used from previous work, published in [4]. All three are relying on hand-crafted features. These three detectors are combined in information fusion with two newly implemented detectors (see Section 5.1.2 and Section 5.1.3 for details). This usage of ensembles of detectors for a complex decision forming has been established as best practice for DeepFake detection. Regarding detection pipelines intended for (forensic) field usage, in [6] the need for fusion-based approaches is strongly argued for as follows: “[…], *a skilled attacker, aware of the principles on which forensic tools work, may enact some counter-forensic measure on purpose* […]. *Therefore, the integration of multiple tools, all designed to detect the same type of attack but under different approaches, may be expected to improve performance, and especially robustness with respect to both casual and malicious disturbances*.”

## 3. Related Work and the Derived Challenge for This Paper

Modern day science means reaching out while standing on the shoulders of giants. In this paper, pre-existing work already extending the German BSI guidelines for IT forensics [3] is used to advance towards a comprehensive concept for modelling media forensic investigation pipelines. Two different branches-related work are considered here: On one hand, the works of Kiltz et al. on evolving the BSI guidelines into the so-called Data-Centric Examination Approach (DCEA) for modern IT forensics (see Section 3.1), and on the other hand, the authors own previous work on a domain adaptation for media forensics (see Section 3.2). At the end of this chapter, in Section 3.3, the challenge addressed in this paper is briefly summarised.

### 3.1. The Data-Centric Examination Approach (DCEA)

As discussed in Section 2.1.2 above, the last published official revision of BSI guidelines dates back to 2011. Since then, it has been used and extended. Significantly updated version, which is also used within this paper, can be found in [25] and is called by its authors the Data-Centric Examination Approach (DCEA). The DCEA re-uses and extends the three core aspects already present in the BSI guidelines from 2011: a model of the *phases* of a phase driven forensic process, a classification scheme for *forensically relevant data types* and *forensic method classes*.

The majority of the extensions done in recent publications focus on domain adaptation for further investigation domains. While the original guidelines focused on hard-disk, RAM and network traffic analysis, [25] extends this scope to also include aspects relevant for digitised forensics (exemplary discussed for the field of dactyloscopy (forensic fingerprint analysis and comparison)). Other publications, like, e.g., [37], adapt to domains with specific constraints like Internet of Things (IoT) forensics.

As a preparatory work for this journal paper, the authors already presented an domain specific adaptation for media forensics, which is discussed in the following section.

### 3.2. Model Adaptation for Media Forensic Tasks

As pointed out above, modelling of media forensics processes is nothing new. In the past, it has mainly been used in academia to provide understandable and reproducible description of media analysis pipelines (see, e.g., [12]). To move forward and address the crucial challenges of development of standards, technical guidelines and certifiable test criteria and test methods as well as research into transparency and explainability of AI driven forensic methods, more elaborate modelling is required.

In [5], a first step for a concept for modelling media forensic investigation pipelines is derived from established guidelines has been done by modelling a corresponding domain adapted data (types) model, derived from DCEA. This new data model, called Media Forensic Data Types (MFDT) is summarised in Table 2.

Taking the typical data streams in media forensics into account, in [5] an adaptation of the existing data models was performed. As starting point the data types from digitised forensics were chosen because they required a less wide-ranging re-modelling than any other previously defined model. The objective for the modelling was (besides the domain adaptation) a specification and overlap-free representation of data types. As a result the following eight media forensic data types (MFDT) were defined: *Digital input data* (MFDT1) considers any kind of media data as it is initially taken as input to the investigation. *Processed media data* (MFDT2) contains all operator output which are media data. *Contextual data* (MFDT3) includes case specific information regarding the investigation process and objects. Contextual data can also be used to control targeted parametrisation, and thus allow case or objects specific parameter optimisation. They also allow for plausibility and fairness evaluations as part of the assessment of an investigation performed. *Parameter data* (MFDT4) contains all configurations and parametrisations for operators in an investigation (except for model data, see MFDT6 below), including those who are used for training of classifiers and models before the actual investigation. *Examination data* (MFDT5) comprises all occurring non-media outputs (e.g., trace information, patterns and anomalies identified) of the investigation. *Model data* (MFDT6) is made up by trained models of machine learning algorithms like rule-based approaches or decision trees as well as models of neural networks (including their network architecture). *Log data* (MFDT7) is a component of the documentation and is used for administration and maintenance (including Syslogs and information about the memory usage). Data in MFDT7 are not relevant for the specific case in the investigation, but are necessary for the administration of the system (e.g., to notice that the memory allocated for the task is not sufficient). *Chain of custody and report data* (MFDT8) characterise the case relevant documentation for integrity and authenticity assurance, as well as the accompanying documentation for the final report. For admissibility in court, the final report would be required following the corresponding chain of custody guidelines. This data model is re-used as it is within this paper as one component in the concept for modelling media forensic investigation pipelines.

### 3.3. The Challenge Addressed in This Paper

The discussions above illustrate the apparent gap between the academic research community (as potential solution providers for forensic methods) on one hand and the requirements imposed onto forensic practitioners on the other hand. The intention of this paper is to facilitate the mutual understanding of these two classes of stakeholders and assist with first steps intended at closing this gap. To do so, first a concept for modelling media forensic investigation pipelines is derived from established guidelines. Then, the applicability of such modelling is illustrated on the example of a media forensic investigation pipeline focusing on the detection of DeepFake videos. At the end of the paper, the benefits of such a planned realisation of AI-based investigation methods are discussed and generalising effects are mapped out.

## 4. Materials & Methods for the Design of a Process-Driven Investigation Model for DeepFake Detection

Even in the most recent academic publications in this field (like [6]), DeepFake detectors are only evaluated in lab tests without any concerns about integration into operational procedures. This might be sufficient for rapid prototyping and academic research, but does not suffice for field applicable forensic methods. In this section, a perspective for the path forward, towards more mature investigations, is presented. Its starts with the necessary methodology and concepts, which are followed by the a discussion on suitable metrics and materials (here specifically an overview over publicly available data sets that exist for benchmarking purposes).

The main methodology for modelling media forensic investigation pipelines was outlined already briefly in [5] (where a domain specific forensic data model was derived) and is significantly extended here.

The ultimate benchmark for any forensic method, which is its applicability in court, can only be achieved on a national level. It is acknowledged here that, due to the fact that all authors are living and working in Germany, the work presented (despite being written in English and presented in an international Journal context) is focused on the German situation and corresponding technical guidelines. Furthermore, at this point it has to be emphasised again that the authors are computer scientists and possesses absolutely no legal training.

Any integration into an operational context would have to focus on various aspects. These would include, among other issues:

- **Organisational:** Specifying the method (as an investigation workflow) and establishing its constraints, limitations and potential errors attached to the method and/or its application.
- **Technical:** Buying and installation of the investigation environment (e.g., forensic workstations) and all required infrastructure (including software such as police casework systems as well as a suitable chain of custody realisation for digital assets).
- **Personnel:** Hiring, training and (re-)certification of experts for applying the method.

Within this paper, the focus lies on the organisational aspects of operationalising investigation methods. It basically follows the BSI guidelines on IT forensics [3] with its split into the separate contexts (the preparation in the forensic process model phase of Strategic preparation (SP), here called *templating*, and the actual usage of a method in the other phases, starting with the Operational preparation (OP), here called *instantiation*).

In the following Section 4.1 the operational units (operators) that are supposed to form parts of an investigation pipeline are modelled. This is followed in Section 4.2 by considerations on the orchestration of operators into an investigation pipeline. Section 4.3 then discusses evaluation best practices and publicly available benchmarking data sets.

The work in this chapter is intended to prepare an illustration of an investigation pipeline for DeepFake detection in Section 5, using own prior work (i.e., detectors).

### 4.1. Modelling of Operator Units

**Figure 2:**
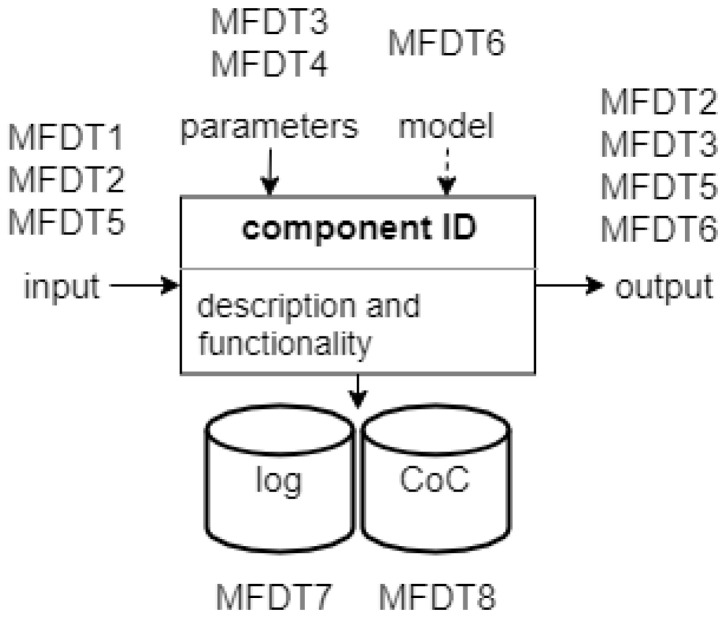
Template structure for a single component (adapted from [5]).

Each operation (or operator) in a forensic process is considered in the approach used here as an atomic processing (black box) component with an identifier, a well defined and documented functionality and (usually) a description of the processing performed in this operation. Each component is modelled here as having four well-defined connectors (see Figure 2): *input*, *output*, *parameters* and *log data*. To pay respects to the particularities of this field and make the following modelling task easier, a fifth connector is defined within this paper for a specific type of operator which requires a knowledge representation or a model for its processing operation. In their case, this fifth connector is labelled *model*. Depending on the nature of the operator this could be a rule set, signature set, statistical model, neural model, or any other form of knowledge representation. Each of these decision-forming approaches has individual advantages and disadvantages. Often, a comparison of these methods and their trained models is solely done based on detection and generalisation performance (e.g., by means of accuracy or area under curve). In addition, sometimes other performance criteria determined are representing feature space dimensionality and number of modelled classes (see, e.g., [38]).

Figure 2 shows the link between media forensic data types (MFDT; see Section 3.2) for the operator description presented above. The input of a component has a form of media data, the court exhibits itself (MFDT1) or after previously done pre-processing steps (MFDT2) or examination data (MFDT5). Depending on the processing step, the generated output could be media data (MFDT2), a derived information on the investigation context (MFDT3) or investigation results (MFDT5). It is also possible during the phase of strategic preparation (SP) that a model is trained (MFDT6). The process control is done by parameters (MFDT4). Furthermore, the gathered contextual data (MFDT3) can be used for optimisation of the parameters in the specific investigation. MFDT3 could, for example, be information about the recording device, resolution or lighting conditions, which might be useful to estimate decision uncertainty and thereby allowing us to estimate the fairness of an investigation. The loading of a model (MFDT6) is limited to model-driven operators, which is why it is shown by a dashed line. Process accompanying documentation will be divided and separately saved in log data (MFDT7) and chain of custody data (MFDT8) based on the modelled data types.

### 4.2. Orchestration of Operators into an Investigation Context

For mature media forensics approaches, the integration (or orchestration) of individual operators into an investigation context has to be done in two distinct episodes: First, in the planning and preparation of a type of investigation in the phase of strategical preparation (SP), and second, in the initialisation of a forensic pipeline for a case-specific investigation in the phase of operational preparation (OP).

First, in SP, the work is focusing on crucial tasks of organisational, personnel and technical nature. Aspects of organisational are, e.g., defining (hereafter called *templating*) workflows and procedures and getting these procedures certified (if necessary). Examples of aspects of personnel nature would be the training of investigators (including their certification if necessary) as well as the assignment of responsibilities. Technical aspects include the hardware and software to be used, i.e., installation of the investigation systems and all required infrastructure (log servers, chain of custody (CoC) infrastructure, etc.) as well as the training of decision models for model driven operators and the benchmarking of trained operator to assess their reliability.

**Figure 3:**
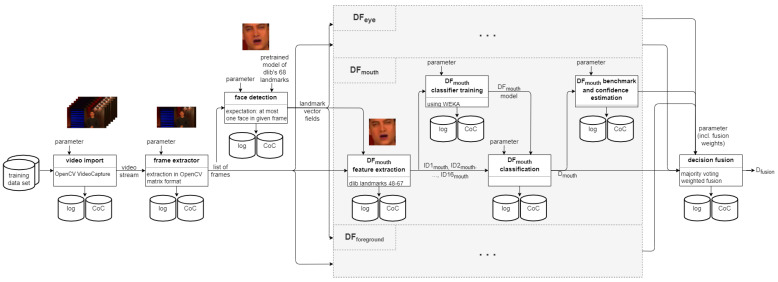
Illustration of the DeepFake detection pipeline described in [4] (exemplified using the first frame of file Celeb-real ID0_0000 [39]) in its **templating** in the forensic process model phase of **Strategical Preparation (SP)**.

At the end of the process in SP, well-specified templates exist that can easily be instantiated into practical investigations as soon as an event/incident triggers an investigation request. Figure 3 shows an example for such a templating, derived from the description of a DeepFake detection pipeline in [4].

The second episode (hereafter called *instantiation*) corresponds to a set of actual investigations, e.g., determining whether a DeepFake manipulation occurred in a video file or stream. Considering the pipeline presented in Figure 1, these investigations cover all phases from OP to Documentation.

**Figure 4:**
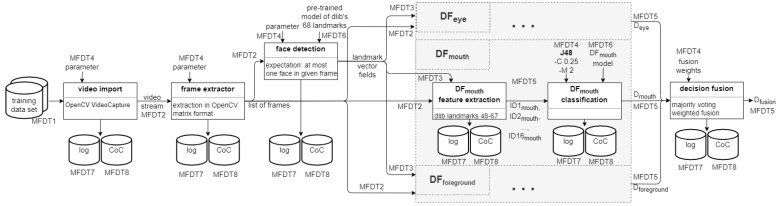
Illustration of the DeepFake detection pipeline from [4], **instantiated** in the forensic process model phase of **Operational Preparation (OP)**, with the inclusion of occurring data types described in [5].

Here, a prepared (as well as benchmarked and potentially certified) template from SP is filled with life by invoking the corresponding orchestration of operators on the assigned processing nodes. Decision models pre-trained in SP are loaded (as shown in Figure 4), together with the used pre-processor and classifier parameters. Thus initialised, the operators are then applied to the input data to the process (MFDT1) to determine traces or information relevant for the investigation at hand.

Since sophisticated analysis pipelines will have to rely on information fusion (i.e., the combination of multiple expert systems; see Section 2.2.2), additionally the required fusion weights required for this purpose have to be loaded from the materials prepared in SP.

In addition to the preparation of the templates for actual investigation pipelines in the SP, corresponding documentation packages are also prepared and constantly updated. When a template is then instantiated for a case in OP, the required documentation packages are marshalled together into the investigation accompanying documentation of the case.

### 4.3. Evaluation Best Practices and Publicly Available Benchmarking Data Sets

As final part of the materials and methods for this paper, in the following sub-sections, first some evaluation best practices are summarised, together with a discussion on suitable metrics, followed by an survey on existing data sets for DeepFake detector benchmarking.

#### 4.3.1. Evaluation Best Practices

As correctly summarised in [6], fusion-based detection methods are required for forensic DeepFake detection to acknowledge the fact that “[…], *a skilled attacker, aware of the principles on which forensic tools work, may enact some counter-forensic measure on purpose* […].” The fusion is therefore not only intended to boost the overall detection performance (at the cost of an higher run-time complexity), but also to improve […] “*robustness with respect to both casual and malicious disturbances*” [6]. While fusion is widely believed to be strongly beneficial to decision problem solution approaches like pattern recognition or anomaly detection, publications like [40] point out that information fusion, which indeed has an huge potential to improve the accuracy of pattern recognition systems, is still very hesitantly applied in the forensic sciences. The reason given is, that a potentially negative impact on the classification accuracy, if wrongly used or parameterised, as well as the increased complexity (and the inherently higher costs for plausibility validation) of fusion are in conflict with the fundamental requirements for forensics. To overcome this hesitation, the typical solution is to:

- Very thoroughly benchmark under different training and evaluation scenarios (see [4]) the individual expert systems (here detectors) to be used in the fusion to precisely establish their requirements and capabilities as well as the error rates attached.
- Benchmark different fusion schemes under different training and evaluation scenarios (see [40]) and establish the impact of different weighting strategies onto the (detection) performance and error patterns.
- Consider decision confidences (where available) into the opinion forming.
- Allow for auditability as well as human oversight for the entire process.

Especially the last item, the aspect of required human oversight is a recent trend for critical AI applications (including forensics) which is, among other regulations, manifested in the current initiative towards an Artificial Intelligence Act (AIA), see [41,42].

A very important issue regarding the benchmarking of pattern recognition-based expert systems is the usage of a fair performance evaluation metric. Here, it is proposed to use the Kappa statistics κ instead of the accuracy. It is basically a single-rater version of Cohen’s Kappa (see [43,44]) in the range [−1,1]. Therefore, the Kappa statistic measures the agreement of prediction with the true class (i.e., the agreement normalised for chance agreement). The following equation shows the computation of the Kappa statistics κ for an n-class problem:

(1) $$\kappa =\frac{1}{n}\sum_{a=1}^{n}\frac{P_{a}-P_{chance}}{1-P_{chance}}$$

For each of the *n* classes, $P_{a}$ is the corresponding percentage agreement (e.g., between the classifier and ground truth) and $P_{chance}$ is the probability of chance agreement. Therefore, κ=1 indicates perfect agreement and κ=0 indicates chance agreement for the overall classification. Only in rare cases negative κ values are achieved, i.e., the classification performance of a system is worse than simple guessing at the class. This is most likely the case when the model was trained to distinguish between patterns completely different than the ones actually presented in the evaluations.

For equally distributed classes, $P_{chance}$ for all classes is simply $\frac{1}{n}$. For differently distributed classes, [44] describes different methods of how to calculate estimate $P_{chance}$. For the computation of the Kappa statistics within this paper, the WEKA implementation [45] is used, estimating Kappa from the distribution of the classes in the supplied test set.

By using Kappa statistics, it is possible to construct for classification-based investigations a degree of closeness of measurements of a quantity to its actual (true) value that is exempt from the influence of the probability of guessing correctly. Such a metric does allow for direct comparison between the classification performances of classifiers on problems of different numbers of classes.

Regarding the interpretability of Kappa κ, ref. [46] presents a mapping between the Kappa value and the agreements of the different raters (see Table 3). Within this paper, the fact is used that it is actually known in the benchmarking performed in SP to which class an input belongs in the evaluations performed. Based on this realisation, here the Kappa values are mapped onto statistical confidence using the mapping defined in Table 3.

The usage of Kappa in research is not without controversy. Authors like Sim et al. [47] argue that: “[…], *the magnitude of kappa is influenced by factors such as* […] *the number of categories* […]”. Furthermore, Kappa is generally not easy to interpret in terms of the precision of a single observation, because according to [48], the standard error of the measurements would be required to interpret its statistical significance. To address this problem, Sim et al. propose in [47] multiple evaluations as the basis for the construction of a confidence interval around the obtained value of Kappa, to reflect sampling errors.

Both facts (implicit influence of the number of classes as well as the standard error in the measurement) are also considered here. In the statistical confidence mapping introduced for this paper, the first fact should be negligible for the practical investigations, because the number of classes considered (and therefore assumedly also their implicit influence) is exactly the same (i.e., two). Regarding the second fact, here the actual classes in the investigations are actually known in the benchmarking performed in SP, which solves part of this problem. Regarding the precision, it is assumed here (based on the achieved evaluation results in initial tests) that it is high enough to allow for meaningful investigations (i.e., the corresponding confidence interval would be suitably small).

In spite of the drawbacks that might be attached to the usage of Kappa, Sim et al. [47] argue that: “*If used and interpreted appropriately, the kappa coefficient provides valuable information on the reliability of data obtained with diagnostic and other procedures* […].”—which is exactly the motivation why Kappa is used in this paper for benchmarking and weight estimation purposes instead of the mere classification accuracy.

#### 4.3.2. Publicly Available (Benchmarking) DeepFake Data Sets

The rapidly growing research efforts for the detection of DeepFakes result in the creation of DeepFake data sets, which are, on the one hand, usable for the implementation and training of new DeepFake detectors. On the other hand, they are needed for benchmarking approaches for detectors. Recent work has shown that data sets are necessary, which include specimen generated with more then one of the generation approaches for DeepFake videos.

In this scope, TIMIT-DF [49] and UADFV [34] were the first publicly available data sets. The number of identities (TIMIT-DF: 43, UADFV: 49) in those databases are very low, as are the perceptual qualities of these DeepFakes. These are the reasons why they have become less relevant during the last years. The Face-Forensics++ [50] data set uses four different creation methodologies (Face2Face, DeepFake, FaceSwap, NeuralTextures). It also increases the amount of DeepFake videos to 1000 per generation algorithm. Note that it is not known to the authors of this paper how many identities have been used to build those 1000 videos.

Not really a part, but provided by the same creators as Face-Forensic++ is the data set Google-DFD [51], which contains 28 identities. It is one more example for a set that includes more than one DeepFake generation approach. Li et al. [39] introduced Celeb-DF with 59 identities. It consists of three parts: Celeb-real (590 videos), YouTube-real (300 videos) and Celeb-synthesis (5639 videos), whereas Celeb-real is used for the DeepFake videos in Celeb-synthesis. Furthermore, the authors in [39] proposed the grouping of several DeepFake data sets into different generations (using the number of frames of the videos in a set): Generation 1 consist of the data sets TIMIT-DF, UADFV and Face-Forensics++. Generation 2 consists of Google-DFD and Celeb-DF. Additionally, the DFDC-Preview data set of Dolhansky et al. [52], which contains of 66 identities, is classified in the second generation of DeepFake data sets, later. This data set is the first part of the DeepFake Detection Challenge (DFDC), which will introduce a newer generation (generation 3) of DeepFakes in [53]. The authors adopt the classification attributes of Li et al. [39]. Every data set which has a total frame amount of 10,000,000 or more as well as 10,000 videos or more is grouped into this third generation. The DFDC data set is another example for a data set that is build with more than one DeepFake generation method. In the time of the publication of Dolhansky et al. [53], there were only two data sets which belonged to this generation: DeeperForensics-1.0 [54] with 100 identities and the DFDC data set of Dolhansky et al. [53] with 960 identities. DeeperForensics-1.0 [54] includes adversarial attacks in DeepFake videos (e.g., added noise, blur, compression), aiming at making detection attempts more realistic (i.e., considering an attacker that tries to hide the traces of the DeepFake attack).

Newer data sets are more and more specific for a defined use case which complicates the grouping of those new databases into the old generations. For example FakeAVCeleb [55] has the size for a generation 3. However, this data set has a different structure. It combines real and fake media as well as image/video and audio data in different ways for 490 identities. It contains video DeepFakes with real audio, audio DeepFakes with real video and also DeepFakes consisting of fake audio and fake video. The real part is reused from VoxCeleb2 [56]. Furthermore, its authors try to increase diversity (in terms of ethnic backgrounds, ages, and gender). DeepFakeMnist+ [57] is a small DeepFake data set which tries to reproduce different emotions or face movements. While in the previously named data sets the DeepFakes are (mostly) created by the data set authors themselves, WildDeepfake [58] consisted of 707 collected DeepFake videos (plus corresponding benign counterpart video) from the internet (i.e., representing an ‘in the wild’ set of mixed/heterogeneous generation methods). The advantage of this set creation strategy is the diversity of different forgery techniques, which are also examined by Zi et al. [58]. Kwon et al. [59] generates 175,776 fake clips from 62,166 real clips with 403 (mostly Koreans) identities with different generation models. They also labelled their data set using categories for *age*, *sex* and *location*. Jain et al. [60] used for the data set DF-Mobio 72 identities. It is also divided into 31,950 real videos and 14,546 DeepFake videos. Note that the real videos are taken from the Mobio data set in McCool et al. [61]. This data set contains videos which are taken by the cameras of mobile devices (i.e., smartphones). Table 4 summarises those data sets regarding to the amount of identities and the real and DeepFake video size.

In addition to these dedicated DeepFake databases, a huge number of publicly available face video databases have also been created in other research domains, which can be used to represent the other class (here, non-DeepFake or genuine face videos). Those data sets can be used to design different training and testing scenarios, to be able to establish facts about the generalisation power of the detectors trained, which is an important aspect of the quality assessment for every method. Such evaluations would have to be performed as part of quality assurance in the strategic preparation (SP) phase of each forensic process.

## 5. Application of the Updated Process Modelling to Describe a Fusion-Based DeepFake Detector

The following two sub-sections summarise the work performed, split into the part done in Strategic Preparation (SP, see Section 5.1) and the one started in Operational Preparation (OP) and then conducted throughout the gathering, investigation and analysis phases of a media forensic process (see Section 5.2).

The test procedures and criteria used here are measurements on the detection performance using Kappa statistics and a discussion of the impact of similarity or dissimilarity of training and test data on the detection performance.

The detection methods used here (and discussed in detail in Section 5.1.2 and Section 5.1.3) are admittedly not amongst the most sophisticated detectors currently available, but the general performance shown, including problems with the generalisation power are representative for the current situation in this field of applied pattern recognition.

### 5.1. Templating (In SP) the Empirical Investigations for This Paper

**Figure 5:**
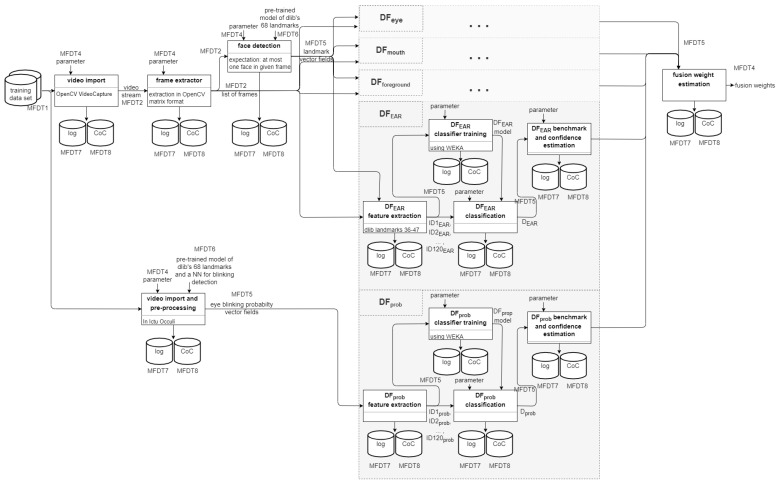
Illustration of the DeepFake detection pipeline **template** (based on Figure 3 above and [4]) created in **Strategic Preparation (SP)** for the usage for the experiments in this paper.

The research performed in [4] is here extended by adding two additional detectors, which have to be included into the benchmarking and fusion accordingly. Figure 5 visualises the additions to the template made in contrast to the original template presented in Figure 3 above.

The detector $DF_{prob}$ has an separate pre-processing pipeline (described in Section 5.1.3 below) while all four other detectors share the same pre-processing pipeline.

#### 5.1.1. Data Sets Used for Training and Benchmarking

Here, from the long list of available data sets (as summarised in Table 4 in Section 4.3.2 above), the same ones are re-used here as in [4] for the necessary training operations of detectors, their benchmarking, the determination of fusion weights and the evaluation of fusion approaches. This is done to keep the results comparable (and is not intended to imply a specific fitness/quality of these sets): The Celeb-DF data set is split into disjointed subsets labelled Celeb-real, Celeb-synthesis and YouTube-real. For training purposes, hereafter referred to as $Celeb_{train}$, Celeb-real and a subset of Celeb-synthesis of 590 videos (which represents the number of samples in Celeb-real) are taken for the training of models. The rest is reserved for the evaluations performed in Section 5.2. In addition, a second benchmarking round is performed using the whole TIMIT-DF data set, consisting of 559 real and 640 DeepFake videos.

#### 5.1.2. Pre-Existing Detectors Re-Used in This Paper

In [4], a total of three different detectors for DeepFake detection were presented, analysing different video areas (eyes, mouth and image foreground). While the detector based on the mouth region showed the best individual results (κ=0.89) in evaluations, the detectors based on eyes and image foreground (κ=0.42) also revealed potential for the detection of DeepFakes with (κ=0.38 and 0.42, respectively).

#### 5.1.3. Detectors Newly Implemented for This Paper

Besides the three detectors from previous work (see Section 5.1.2), two new detectors (one hand-crafted and one neural network based) aiming at DeepFake detection based on eye blinking inconsistencies are proposed here. For both the pre-processing is performed frame by frame. First, the face is detected using dlibs 68 landmarks [66], with each eye represented by 6 key points [4]. For comparison purposes, the facial region is resized to an area of 256 × 256 pixels. For the hand-crafted approach, the so-called eye-aspect ratio (EAR), given by $EAR=\frac{|p_{2}-p_{6}\left|\right|+\left|\right|p_{3}-p_{5}\left|\right|}{2||p_{1}-p_{4}\left|\right|}$ [35], is calculated as the first representation for each eye ($EAR_{l}$ and $EAR_{r}$).

For the neural network-based approach, the model presented in [31] by Li et al. is used. To define the degree of aperture of each eye, the bounding box given by the corresponding 6 landmarks is determined. This is followed by probability estimation of the eye blinking based on a Long-term Recurrent Convolutional Network (LRCN), using the bounding box of each eye as input. As a result of both pre-processing approaches, two vectors (one for each eye) are given, representing the EAR and probability of eye blinking, respectively.

**Figure 6:**
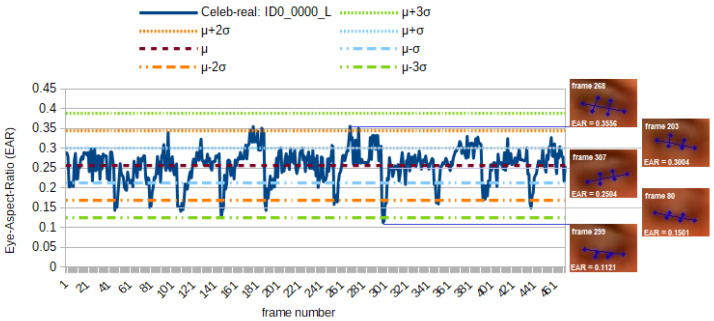
Illustration of the eye-aspect ratio (EAR) and the proposed descriptors (eye aperture analysed for the left eye of file Celeb-real ID0_0000 [39], the small sub-figures on the right hand side showing the corresponding aperture using segments of selected frames of that video).

For the following classification based on these pre-processed signals, only a hand-crafted approach is taken due to time constraints. In addition to $EAR_{l}$ and $EAR_{r}$, two additional representations of the blinking behaviour as difference quotient ($diff_{l}\left(i\right)=EAR_{l}\left(i\right)-EAR_{l}\left(i-1\right)$ and $diff_{r}\left(i\right)=EAR_{r}\left(i\right)-EAR_{r}\left(i-1\right)$) for each eye are computed using consecutive video frames (i) and (i−1). Based on [35], the values for the following statistical descriptors μ (arithmetic means), μ−σ, μ−2σ, μ−3σ, μ+σ, μ+2σ, μ+3σ are calculated, where σ is an a posterior determined standard deviation. In Figure 6, those descriptors are plotted as horizontal lines. Using these seven different descriptors, two different types of features are derived: noise estimators (here, a set of crossing rates) and energy estimators (here, equivalent to the area under a curve, representing the aperture of the eye). Additional features are derived trying to iteratively estimate the skew of the distribution (which has to be assumed since the eye is usually opened for a longer time than it is closed do to blinking). Including (μ) and (σ), this results in a set of 30 features that are gathered for each of the four representations. Then, the four sub-vectors are concatenated in a fixed sequence, which in turn results in a vector of 120 features length.

What can be derived from Figure 6 is the fact that hand-crafted feature designs, like the ones discussed above, can very well represent semantic characteristics of the signal (here, the blinking of an eye in a video stream) with easily interpretable features.

The classification models used are acquired by training five different machine learning algorithms, specifically NaiveBayes [67,68], LibSVM [69], SimpleLogistic [70,71,72], JRip [73,74] and J48 [75,76] (the WEKA implementations of these pre-existing algorithms in their standard configurations; note: these classifiers were selected by the authors on basis of previous work, for future work, more sophisticated classifier selection and parameter optimisation could be required), of which the best one in terms of κ value is selected for the investigation process template created. In the comparison of these five different machine learning methods, no significant differences in run-time for the model training was detectable. Only with respect to detection performance, given using Kappa statistics κ, differences are noticeable. Table 5 shows the performances of each model and highlights the best one per detector, which then is used in the evaluations performed.

#### 5.1.4. Fusion Operators and Weight Estimation

In [4] a total of five different fusion strategies (feature-level fusion as well as decision-level in forms of simple majority voting and weighted-average fusion) have been shown to increase the performance over single detectors. In line with this approach, the strategies simple majority voting and weighted fusion are re-used here, but in contrast to [4] the weights are determined in SP phase using κ instead of the decision accuracy. As discussed in Section 4.3.1, Kappa is a more fair approach regarding unevenly split data sets and, as shown in Section 4.3.2, most public available data sets have a higher amount of DeepFake videos. Since there are five detectors, the weights have to be adjusted based on the results on the training data set $Celeb_{train}$ resulting in $w_{eye}=0.1590$, $w_{mouth}=0.3724$, $w_{foreground}=0.1757$, $w_{EAR}=0.1548$ and $w_{prob}=0.1381$. A detailed comparison of the individual Kappa values for each individual detector and its derived weights can be found in Table 6.

As proposed in Section 4.3.1, a detector should only be included if at least a significant confidence mapping is achieved. For forensic field applications this value should obviously be well above κ=0.95, in academic research it should be at least in the range of “fair to good” with κ≥0.4.

Here, for the sake of illustrating the effect of this decision, this rule is violated on purpose. As a result the detector $DF_{eye}$ as well as the two new detectors $DF_{EAR}$ and $DF_{prob}$ are included in the template as inputs to the fusion despite the fact that their performances in benchmarking are below κ=0.4.

### 5.2. Instantiation of the Pipeline for the Evaluations in This Paper

**Figure 7:**
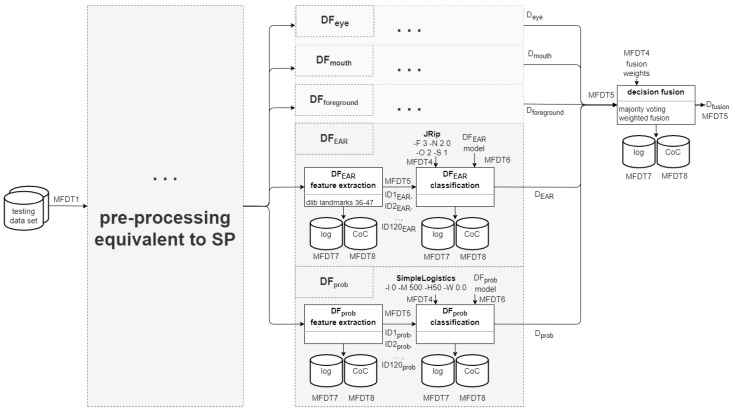
Illustration of the DeepFake detection pipeline in this paper, based on [4], **instantiated** in the forensic process model phase of **Operational Preparation (OP)**, with the inclusion of occurring data types described in [5].

Figure 7 shows the instantiation of the pipeline in OP for the evaluations performed in this paper. In contrast to Figure 4, it shows the extension made since [4] (i.e., the addition of two additional detectors). Since the whole pre-processing is equivalent to the one performed in SP (see Figure 5), this part is omitted here.

#### 5.2.1. Data Sets Used for Evaluation

Here, from the long list of available data sets (as summarised in Section 4.3.2 above), the following are reserved for the evaluation operations of the individual detector, YouTube-real (300 real videos) and a part of Celeb-synthesis (5049 DeepFake videos), this set $Celeb_{Test}$ is disjointed from the data used for training, benchmarking and weight estimation purposes in Section 5.1 above. In addition, the benchmarking for the fusion is done on the same data set as in [4] and contains a total of 120 real and 120 DeepFake videos of Celeb-DF.

#### 5.2.2. Single Detector Evaluation Results

**Figure 8:**
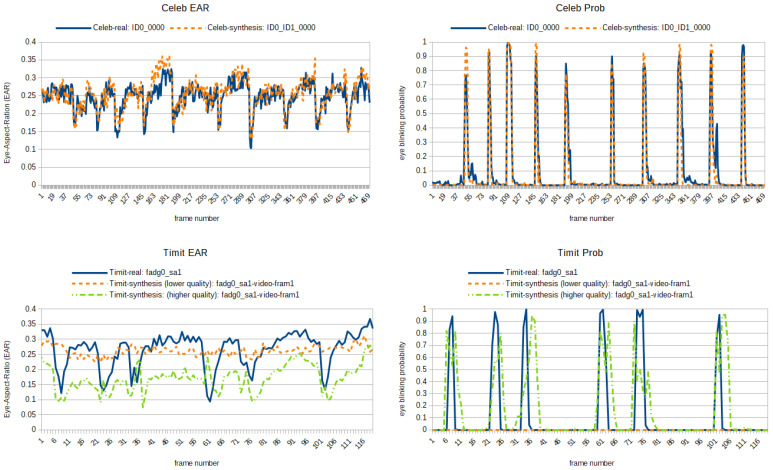
Comparison of the acquired blinking curves for a DeepFake and its source based on EAR (**left**) and probabilities (**right**) for both considered data sets Celeb-DF (**top**) and TIMIT-DF (**bottom**).

The evaluation of the $Celeb_{Test}$ shows in Table 7 a drastic decrease in Kappa for both $DF_{EAR}$ and $DF_{prob}$, reducing it to almost zero. To further investigate the causes, the corresponding curves for a DeepFake video and its corresponding source are compared. As shown in Figure 8 (top row), the curves for both detectors appear to be almost identical, showing that the synthesis method used in Celeb-DF is able to reproduce the natural blink behaviour.

The tests on TIMIT-DF show a different result, both in terms of generated curves as well as Kappa values. Both $DF_{EAR}$ and $DF_{prob}$ show Kappa above 0.4, with 0.55 and 0.43, respectively. In addition, there are also major differences in the curves to be seen in Figure 8, and thus showing that the DeepFake blinking behaviour in this data set is clearly distinguishable from real blinking. The comparison of higher and lower quality DeepFakes of TIMIT-DF further shows that the higher quality is closer to a real blink. Taking into account the generations in which both data sets are placed, it can either be seen as a potential flaw in earlier generations or it could be caused by the generation method (which is not considered in the generations specified).

#### 5.2.3. Results of the Fusion-Based Detection

As shown in Table 8, the inclusion of the new detectors results in a slight drop in the average detection performance. For the majority voting, κ=0.542 is determined, in contrast to the previously achieved performance of κ=0.725, a drop in performance of 0.18. Presumably, this can be explained by the use three of the five detectors individually showing κ<0.4 in $DF_{eye}$, $DF_{EAR}$ and $DF_{prob}$, all based on eye features outvoting the other two (performance-wise better) detectors.

In the case of the weighted fusion, the drop is smaller, but still recognisable (by 0.02 from 0.808 to 0.783). The optimal decision threshold for the fusion operator is determined iteratively here. It is noticed in these experiments that a shift in optimal threshold value for the classification shifts from 0.65 to 0.5 occurs. This new threshold could be equivalent to $DF_{mouth}$ (with a weight of 0.3724) agreeing with at least one other detector, which is very similar to the fusion outcome shown in [4].

## 6. Results

This chapter provides a brief summary of the results, before the following Section 7 projects the conclusions onto the contributions identified in Section 1.

### 6.1. Experimental Evaluation Results and Comparison with the SOTA/Related Work

It is apparent that the detection performances of the detectors used in this paper are not fit to compete with the best detection performances presented in the state-of-the-art publications on detector designs. However, it can be assumed that the findings presented here generalise as follows:

- The comparison of the investigation results and the differences experienced when looking at the performances on the TIMIT-DF and Celeb-DF data sets indicate a sensitivity of trained detection approaches to specific DeepFake generation methods. In consequence two alternative strategies for compensating this sensitivity should be explored: Generalisation or specialisation of the training scenario for detectors. For the first alternative, training sets with large heterogeneous DeepFake parts would be required, potentially resulting in models with a high false positive rate due to the fact that the model component(s) characterising the DeepFake class are very dispersed in the feature space. For the second alternative, targeted training for the different DeepFake generation would be required, effectively transforming the task into an *n*-class problem.
- Extensive benchmarking of detectors is required for any application of forensic methods. What is true for single detectors, becomes even more relevant when combining single expert systems into a fusion approach. The practical evaluations summarised in Section 5.2 above show how adding two detectors, which are performing individually better than the probability of guessing correctly (which would be κ=0), negatively impairs a fusion outcome. What has not been reflected upon in the discussions made in Section 5 is that the question of fairness and bias are also becoming much more complex in the context of fusion: Out of the five detectors used within this paper, three are concentrating on the eye regions. This effectively leverages the weight estimation for fusion weights, which were made under the implicit assumption of the independence of involved detectors.

### 6.2. Lessons Learned during the Templating and Instantiating of the Pipeline in SP/OP

When reflecting the work presented in this paper on the three specific aspects for current research needs according to the whitepaper “Secure, robust and traceable use of AI-problems, procedures and actions required” [1] as discussed in Section 1, the following can be summarised: Instead of focusing on research on effective countermeasures (i.e., DeepFake detectors), like most of the scientific papers currently published, the work presented is focusing on the other two aspects: first, supporting the development of standards, technical guidelines, test criteria and test methods as well as, second, the research into methods of transparency and explainability.

The efforts invested in the Strategic Preparation (SP) of a forensic process are assumed to prepare for effective response in case of an incident. They are intended to increase forensic readiness of response and investigation units as well as strengthening the whole field by providing standardised (and certified) methods and procedures.

Reflecting this basic principle of a hard split into SP and operations (OP, DG, DI, DA and DO) into academic research might seem weird at first, but is, in the opinion of the authors, a step that might in the long run help to bridge the gap between academic research in media forensics and the practitioners requiring court admissible methods.

What this split is supposed to provide are more precise process descriptions, which can easier be verified by third parties. Furthermore, they make training, benchmarking and testing procedures more transparent and are thereby supposed to better allow the identification of influence factors, training bias and potential error sources.

In addition to the templating and instantiation considerations regarding the design and implementation of processes, the modelling work presented has a second relevant aspect: the domain-adapted media forensic data types presented in Table 2. With their help, the actual data flows in complex systems, such as police case management systems, should become manageable. More details regarding this data model, its usage and benefits are presented in [5].

## 7. Conclusions and Discussion

Drawing the conclusions from the work presented and projecting them to the contributions identified in Section 1, it has to be said that:

The **need for modelling forensic processes** is reasoned upon, with a brief overview over forensic process modelling requirements and some best practices for media forensics (in Section 2.1). It can be (and is, e.g., in [1]) summarised as: (a) the development of standards, technical guidelines, test criteria and test methods; (b) research into effective detectors/countermeasures and (c) research into methods of transparency and explainability of AI-driven methods. Out of these three well-grounded needs, current research (esp. academic research) in media forensics focuses mainly on (b), ignoring the fact that without also achieving the other two, the required degree of maturity for court room acceptability will not be achieved. This paper tries to highlight this gap and facilitate the understanding between the media forensic research community and practitioners in the field of applied forensics.

A **concept for modelling media forensic investigation pipelines** is derived from established guidelines. Due to the nationality of the authors, this concept is derived from long standing German guidelines on IT forensics, which are extended here to better fit the specifics in the field of media forensics. By doing so, the authors do not claim that this starting point, published by the German Federal Office for Information Security (BSI) in 2011, is the most suitable choice (which it obviously is not), but acknowledge the fact that regulation concerning the admissibility of procedures and methods happens on a national level. The introduced approach to modelling investigation pipelines focuses on a two-step procedure: First, in a preparatory step called here Strategic Preparation (SP), the planning or *templating* of an investigation pipeline, combined with all organisational, technical and personnel steps required for implementing one or multiple pipelines of this nature (see Section 4.2) happens. The operations in this phase would include among other things the certification or investigation methods and procedures as well as the training of corresponding experts. In the following step, here called Operational Preparation (OP), the previously prepared pipelines are *instantiated* as required, i.e., used in a standardised way to perform specific investigations.

Despite the fact that the work presented in this paper is still a rough sketch on the actual work required to get methods ‘court ready’, it gives an idea on the required next steps after a technical solution (e.g., detector) has been found fit for publication by its authors.

The **applicability of the introduced modelling is illustrated** on the example of a media forensic investigation pipeline focusing on the detection of DeepFake videos, extending previous work of the authors on possible fusion-driven detection pipelines. The results show after adding two further detectors, which where in benchmarking in SP on purpose wrongfully determined to be suitable (κ∼0.4), a drop of the detection results in the experiments. This implies that the benchmarking strategies used here still leave significant room for improvement.

The **benefits of such a planned realisation of AI-based investigation methods** are discussed to some extend. Here, it is apparent that these discussions only cover the tip of the iceberg! One recent trend of how to counter the issue of manipulations is well summarised in [6] by the following statement: “*Face manipulation brings an array of complex legal issues. There is no comprehensive legislation on the use of manipulated images, yet several aspects are already regulated in various countries. It should hence not surprise that the development of new manipulation technology and the detection thereof also leads to new issues and questions from a legal perspective which deserve further research. If it is used to mislead, manipulated images can cause significant harm* […] *In some countries, altered (body) images used for commercial purposes (such as the fashion industry) need to be labelled. More generally, legislative proposals in several countries try to tackle the transparency issue by imposing an obligation to inform users that they interact with AI-generated content (such as DeepFakes)*”. However, this implicitly only white hat application of methods like DeepFakes. No (criminal or other) threat actor will adhere to such an obligation when spreading fake news or other media-related manipulations. As a consequence, entities such as news agencies strongly relying on media objects submitted from external sources would also require mature manipulation detection mechanisms that would have to be integrated into their already established source (material) verification routines. The exact extent and scope of such analysis methods and ‘filters’, their transparency and fairness, as well as their potential impact to public and politic debates are currently a hot debate especially in Europe (see for example [77] for the discussion of free speech implications of Article 17 (regulating upload filters) of the EU ‘Directive on copyright and related rights in the Digital Single Market’ as adopted in 2020).

## 8. Future Work

Instead of focusing on research on effective DeepFake detectors, the work presented is concentrating on the two aspects of supporting the development of standards, technical guidelines, test criteria and test methods as well as the research into methods of transparency and explainability. Despite the fact that the detectors in this paper were mostly used for illustrative purposes, their quality of course also has to be enhanced, either by improving the existing detectors or including better ones into the fusion-driven decision system. The next step along these empirical lines would then be the design of best-practices for evaluations, focusing on data sets, first allowing for more realistic error rate estimates (e.g., ‘ìn-the-wild’ sets (eventually also including counter-forensics), like [53,54,58]) and second for fairness considerations (e.g., looking at challenging data sets like [55,59] to determine racial bias). As shown in Table 4, a wide range of suitable data sets is currently available for such a purpose.

In addition to those single-classifier benchmarking aspects, also bias evaluations regarding the fusion would be necessary to be performed: In our illustrative example pipeline, the five detectors were chosen and combined in a way that is overvaluing/biased towards parts of the signal (here, the eye regions the video-which is significantly over-represented with three out of the five detectors focusing on this small part of the video) effectively counteracting the actual weighting done for the fusion. This might first seem a unlikely situation in practice but with many neural network driven detection methods, it remains unclear what exactly the features learned are actually representing. Therefore, such situations are a threat that is, in the opinion of the authors, likely to occur with learned feature spaces and that is so far going mostly unnoticed by many practitioners. As a consequence it would be required to understand such learned feature spaces to avoid such kind of bias.

Returning to the infrastructure considerations dominating the work in this paper, two separate aspects are discussed below: first, perspectives for extending the presenting modelling and evaluation work, and second, the big issue of demystifying modern machine learning and AI systems.

### 8.1. Extending the Presented Modelling and Evaluation Work

As indicated by the results discussed in Section 7 regarding the perceived drop in the fusion performance after the adding of two assumed good new detectors, one of the most important next steps regarding the introduced approach is the design of a **practical benchmarking framework for single detectors in Strategic Preparation (SP)**. This would allow for a more fine-granular detector evaluation and corresponding fusion operator design and parameterisation (including the fusion weights). Such a benchmarking framework would have to consider a wide range of data sets, classified using the four different concepts for DeepFake generation (facial reenactment, facial replacement (or face swapping), face editing and face synthesis) as well as clearly identified types of traces imposed to the media objects by the corresponding modifications. Only with such a framework, necessary reasoning regarding performance influencing factors as well as bias and/or fairness issues (see e.g., [55,59]) can be performed.

In addition to the escalation of the benchmarking extent, also the basic strategies should be revised. Here, in accordance with the work presented in [60], **a re-modelling of the detection/classification problem as an *n*-class problem** (where *n* corresponds to the number of different DeepFake creation strategies, see Section 2.2) might become necessary. This paradigm shift is assumedly strongly beneficial to the detection performance as well as the interpretation of error behaviours (i.e., the decision plausibility and transparency). Conceptually, it allows us to handle each of the different creation strategies as what they are: different manipulation pipelines leaving clearly distinguishable artefacts or traces. Technically, it would allow to train much more precise decision models for each DeepFake creation strategy, instead of representing them all as different sub-spaces of the class ‘DeepFake’ in the currently trained models.

A second important issue for future work is the extension of **investigations into error, loss and uncertainty in the forensic processes** as motivated in previous work (esp. [25]). This requires research efforts especially in the field of demystifying AI system decisions (see Section 8.2 below), not only for classical decision methods with hand-crafted features, but also for the more recent approaches relying of neural network to learn feature spaces that lack intuitive interpretation.

Third, but most important, increasing the maturity of approaches requires the **extension of the work from modelling into practical frameworks**. Here, joint efforts with system developers (e.g., for police case management systems) as well as certification bodies would be required to achieve this goal. An very interesting success story in this regard is the following: Regarding digital camera forensics a major breakthrough can be seen in the law case *United States of America v. Nathan Allen Railey* (United States District Court for the Southern District of Alabama (for a short summary of the relevant part of the court case see [78])). In the Daubert hearings of this case, the method of digital camera authentication based on intrinsic characteristics of its image acquisition sensory developed by Jessica Fridrich and her university research group (see e.g., [79]) got accepted for the first time as forensic evidence. The FBIs Forensic Audio, Video, and Image Analysis Unit (FAVIAU) established in the Daubert hearings that this approach (and the corresponding tool ‘FindCamera’ developed and evaluated in a public private partnership effort lead by the FBI and the US Airforce Research Labs) meets all necessary Daubert criteria and the presiding judge furthermore decided that this evidence (or more precisely the FBI expert testimony based on this media forensic analysis) also meets the FRE rule 702 criteria.

As discussed above in Section 2.1.2 forensics are entirely governed by national legislation. Therefore, this requires nation specific efforts to get such methods and procedures court-ready.

### 8.2. Demystifying Machine Learning and AI Systems

To demystify machine learning, a comparison of the advantages and disadvantages of each individual decision forming method as well as each trained model is required. Besides the previously mentioned aspects of detection and generalisation performances and the dimensionality and composition of the feature space, further aspects such as training duration (including training success estimates) and model complexity (including its impact on explainability) have to be considered. Modern neural network based analysis and detection methods show impressive results regarding detection results on data similar to the training data used to create the network. What are still issues for research are the generalisation power of such methods (i.e., how well the systems perform on previously unseen data) as well as the transparency, correctness and fairness of their decisions.

Recent research is looking especially into these questions of understanding the inner workings of black box neural networks, e.g., by determining the most important neurons in a network and deducing knowledge from those analyses. Some papers, like [80], even extend into automated linguistic annotation methods to provide better understandable descriptions of internal workings.

## Figures and Tables

**Table 1:** Sets of examination steps for digital forensics as defined in [25] (updated from [3,26]).

Phases	Description (According to [25])
Strategic preparation (SP)	Includes measures taken by the operator of an IT system and by the forensic examiners in order to support a forensic investigation prior to an incident
Operational preparation (OP)	Includes measures of preparation for a forensic investigation after the detection of a suspected incident
Data gathering (DG)	Includes measures to acquire and secure digital evidence
Data investigation (DI)	Includes measures to evaluate and extract data for further investigation
Data analysis (DA)	Includes measures for detailed analysis and correlation between digital evidence from various sources
Documentation (DO)	Includes measures for the detailed documentation of the proceedings, also for the transformation into a different form of description for the report of the incident

**Table 2:** Media Forensic Data Types (MFDT) proposed in [5].

Data Type	Description
MFDT1 Digital input data	The initial media data considered for the investigation.
MFDT2 Processed media data	Results of transformations to media data (e.g., greyscale conversion, cropping)
MFDT3 Contextual data	Case specific information (e.g., for fairness evaluation)
MFDT4 Parameter data	Contain settings and other parameter used for acquisition, investigation and analysis
MFDT5 Examination data	Including the traces, patterns, anomalies, etc that lead to an examination result
MFDT6 Model data	Describe trained model data (e.g., face detection and model classification data)
MFDT7 Log data	Data, which is relevant for the administration of the system (e.g., system logs)
MFDT8 Chain of custody & report data	Describe data used to ensure integrity and authenticity (e.g., hashes and time stamps) as well as the accompanying documentation for the final report.

**Table 3:** Kappa values, agreements according to [46] and the statistical confidence mapping used in this paper.

Kappa Value κ	Agreement According to [46]	Confidence Mapping Used Here
κ<0	No agreement	Poor
0≤κ<0.2	Slight agreement	Poor to fair
0.2≤κ<0.4	Fair agreement	
0.4≤κ<0.6	Moderate agreement	Fair to good
0.6≤κ<0.8	Substantial agreement	
0.8≤κ≤1.0	Almost perfect agreement	Good

**Table 4:** Overview on existing publicly available video reference data sets for DeepFake detection (in case of an ‘?’ in the table, the number of individuals has not been documented for this data set). The TIMIT-DF and Celeb-DF data sets used in this paper are marked in bold.

Data Set	# Individuals	# Real Video	# DeepFake Video
UADFV [34]	49	49	49
**TIMIT-DF** [62,63]	43	559	640
FaceForensics++ [50,64]	?	1000	4000
DFD [51]	28	363	3068
**Celeb-DF** [39]	59	890	5639
DFDC [53]	960	23,654	104,500
DeeperForensics [54]	100	50,000	10,000
WildDeepfake [58,65]	?	3805	3509
DeepFakeMnist+ [57]	?	10,000	10,000
FakeAVCeleb [55]	490	20,000+	20,000+
KoDF [59]	403	62,166	175,776
DF-Mobio [60]	72	31,950	14,546

**Table 5:** Achieved κ values for each classification model using $Celeb_{Train}$ in 10-fold cross validation.

Detector	NaiveBayes	LibSVM	Simple Logistics	JRip	J48
** $DF_{EAR}$ **	0.0695	0.3254	0.3508	**0.3678**	0.2966
** $DF_{prob}$ **	0.2162	0.0063	**0.3275**	0.2480	0.2273

**Table 6:** Overview of the results of each individual detector and the derived fusion weights.

Detector	κ	Fusion Weight
$DF_{eye}$ [4]	0.38	$w_{eye}=0.1590$
$DF_{mouth}$ [4]	0.89	$w_{mouth}=0.3724$
$DF_{foreground}$ [4]	0.42	$w_{foreground}=0.1757$
** $DF_{EAR}$ **	0.37	$w_{EAR}=0.1548$
** $DF_{prob}$ **	0.33	$w_{prob}=0.1381$

**Table 7:** Overview of the results for the proposed individual detectors on the specified testing data set.

Detector	κ on ${\mathrm{Celeb}}_{\mathrm{Test}}$	κ on TIMIT-DF
$DF_{EAR}$	0.0191	0.552
$DF_{prob}$	0.0408	0.433

**Table 8:** Overview of the results for the fusion strategies in comparison to previously achieved results given in [4].

Fusion Strategy	κ
majority voting (old detectors) [4]	0.725
weighted fusion (old detectors) [4] threshold = 0.65	0.808
majority voting (5 detectors)	0.542
weighted fusion (5 detectors) threshold = 0.5	0.783

## Data Availability

In this work the following the pre-existing reference databases have been used for our evaluations: TIMIT-DF [49,62] and Celeb-DF [39]. They are publicly available at: https://www.idiap.ch/en/dataset/deepfaketimit (accessed on 15 April 2022), https://github.com/yuezunli/celeb-deepfakeforensics (accessed on 15 April 2022).
